# Lipid Profile and Apolipoprotein B Serum Levels in the Vietnamese Population With Newly Diagnosed Elevated Low-Density Lipoprotein Cholesterol and Association With the Single-Nucleotide Variant rs676210: Cross-Sectional Study

**DOI:** 10.2196/76850

**Published:** 2025-08-07

**Authors:** Quyen Thuy Nguyen, An Viet Tran, Bao The Nguyen, Hoa Thai Nguyen, Nhung Thi Hong Thai, Hen Huu Phan

**Affiliations:** 1 Faculty of Medicine Can Tho University of Medicine and Pharmacy Can Tho Vietnam; 2 University of Medicine and Pharmacy Hue University Thua Thien Hue Vietnam; 3 Cho Ray Hospital Ho Chi Minh Vietnam

**Keywords:** APOB rs676210 polymorphism, APOB, ApoB, apolipoprotein B, lipid profile, elevated LDL-C, low-density lipoprotein cholesterol

## Abstract

**Background:**

Apolipoprotein B (APOB) rs676210 polymorphism has been associated with altered lipid metabolism and cardiovascular risk in various populations; however, data from Vietnamese populations remain limited.

**Objective:**

This study aimed to investigate the association of the APOB rs676210 variant with lipid profiles among Vietnamese individuals newly diagnosed with elevated low-density lipoprotein cholesterol (LDL-C).

**Methods:**

A cross-sectional study was conducted among 69 Vietnamese adults newly diagnosed with elevated LDL-C (≥130 mg/dL) at a tertiary hospital in Southern Vietnam. Participants were genotyped for APOB rs676210 using real-time polymerase chain reaction (PCR) with allele-specific probes. Lipid profile components, including LDL-C, high-density lipoprotein cholesterol (HDL-C), non–HDL-C, and ApoB, were compared across genotype groups (AA vs GA/GG) and alleles (A vs G). Statistical analyses involved t tests, chi-square tests, and multivariable linear regression adjusted for age, sex, the BMI, and diabetes. *P*<.05 was considered statistically significant.

**Results:**

Of the 69 participants, 32 (46.4%) carried the AA genotype, while 37 (53.6%) carried the GA or the GG genotype. The AA genotype was associated with significantly higher LDL-C (mean 5.19, SD 0.95, vs mean 4.37, SD 0.97, mmol/L; *P*<.001), non–HDL-C (mean 5.94, SD 1.08, vs mean 5.31, SD 1.22 mmol/L; *P*=.03), and ApoB (mean 149.5, SD 26.3, vs mean 136.9, SD 15.2, mg/dL; *P*=.02) and lower HDL-C (mean 1.26, SD 0.31, vs mean 1.44, SD 0.39, mmol/L; *P*=.03) compared to the GA/GG genotype. Allele-based analysis showed that carriers of the A allele (98/138, 71%) also had higher LDL-C (mean 4.91, SD 1.02, vs mean 4.36, SD 0.97, mmol/L; *P*=.004) and ApoB (mean 145.6, SD 23.2, vs mean 135.9, SD 16.0, mg/dL; *P*=.02) than G allele carriers (40/138, 29%). These associations remained significant after multivariate adjustment.

**Conclusions:**

APOB rs676210 polymorphism is associated with significant differences in lipid profiles among Vietnamese adults with elevated LDL-C. Specifically, the A allele and the AA genotype confer a more atherogenic profile, suggesting potential utility as a genetic marker in lipid screening and personalized cardiovascular risk management in this population.

## Introduction

Cardiovascular disease (CVD) remains a leading cause of mortality worldwide, and dyslipidemia is a key modifiable risk factor contributing to this burden. Elevated low-density lipoprotein cholesterol (LDL-C), in particular, is a significant causal factor in atherosclerotic cardiovascular disease (ASCVD) [1]. In many Asian populations, including Vietnam, the prevalence of lipid disorders is high, with roughly one-third to nearly half of adults meeting the criteria for elevated LDL-C levels [2]. In this context, there is a growing body of research in Vietnam focusing on genetic factors related to CVD, highlighting the emergence of genomics as a relevant field [3-5]. Understanding genetic contributions to lipid abnormalities in Vietnamese patients is thus increasingly important to improve cardiovascular risk stratification and guide personalized interventions.

Apolipoprotein B (ApoB) is the primary protein component of LDL and other atherogenic lipoproteins, and it plays a crucial role in the assembly, transport, and cellular uptake of these particles. ApoB is a major structural protein of very low-density lipoproteins (VLDLs) and low-density lipoproteins (LDLs), mediating their interaction with cellular receptors [6]. Given its central function, genetic variations in the *APOB* gene can significantly impact lipid metabolism. Indeed, numerous single-nucleotide polymorphisms (SNPs) in *APOB* have been associated with altered plasma lipid levels and increased atherosclerosis risk [7]. One such polymorphism is rs676210, a c.8216G>A variant in exon 26 of *APOB* that results in a proline-to-leucine substitution at codon 2739 (p.Pro2739Leu) [6]. This missense mutation is of particular interest biologically, as it is expected to induce a functional change in the ApoB-100 protein structure. The variant has been reported to influence LDL particle characteristics; for example, rs676210 has been linked to the susceptibility of LDL to oxidative modification [8]. Oxidized LDL has a pathogenic role in plaque formation; thus, such a genetic effect could directly affect ASCVD risk.

Epidemiologically, prior evidence suggests that rs676210 may be relevant to interindividual differences in lipid profiles and coronary risk. Genome-wide analyses have identified rs676210 as a locus associated with plasma lipoprotein traits [8,9]. Notably, the minor (A) allele of rs676210 was associated with a more favorable lipid profile (lower total cholesterol [TC], triglycerides [TGs], and LDL-C and higher high-density lipoprotein cholesterol [HDL-C]) in a large cohort study [8]. In addition, this SNP has been implicated in cardiovascular outcomes: for instance, it was associated with myocardial infarction risk in Chinese populations, likely mediated by hyperlipidemia and higher ApoB levels [10,11]. However, findings across studies have not been consistent, and data on rs676210 are scarce in Southeast Asian groups, such as the Vietnamese.

In Vietnam and other middle-income countries, resources for comprehensive genotyping (eg, whole-genome sequencing) are limited [12]. Therefore, focusing on common functional SNPs, such as rs676210, is a practical approach to investigate genetic predisposition to dyslipidemia in these populations. Given the rising burden of hypercholesterolemia in Vietnam [13], it is pertinent to investigate whether genetic polymorphisms, such as *APOB* rs676210, contribute to interindividual variations in LDL-C levels and related lipid indices among the Vietnamese population. In contrast, statin therapy has been shown to be effective in improving lipid profiles in high-risk patients [14,15]. However, the response to statins can vary considerably among individuals, possibly due to underlying genetic factors [6,10]. In this context, the identification of lipid-related polymorphisms may provide a useful foundation for tailoring preventive and therapeutic interventions. This study thus aimed to explore the association of rs676210 with plasma lipid parameters (LDL-C, non-HDL-C, HDL-C, and ApoB levels) in a cohort of Vietnamese patients newly diagnosed with elevated LDL-C. By doing so, we sought to clarify the biological and clinical significance of this variant in an Asian middle-income country, where identifying key genetic markers could aid in improving the screening and management of dyslipidemia.

## Methods

### Study Design and Population

This cross-sectional descriptive study was conducted among adults undergoing routine health checkups at Can Tho University of Medicine and Pharmacy Hospital, a major medical center in Can Tho City, the economic, cultural, and health care hub of the Mekong Delta region in Southern Vietnam. The study was implemented from April 2023 to February 2025. Participants were identified during annual occupational health examinations and were newly diagnosed with elevated LDL-C. None had received lipid-lowering therapy prior to enrollment.

A nonprobability convenience sampling method was used. Eligible participants were individuals aged 18 years or older who were diagnosed with elevated LDL-C, defined as LDL-C≥130 mg/dL (3.4 mmol/L), based on the previous literature and partly because the National Cholesterol Education Program (Adult Treatment Panel III), or NCEP ATP III, guidelines have identified this threshold as predictive of an increased risk of ASCVD [16-18]. Strict exclusion criteria were applied to ensure participant safety and homogeneity of the study population. Patients who were currently taking medications known to affect blood lipid levels, such as corticosteroids, immunosuppressants, oral contraceptives, and CYP3A4 inhibitors (including diltiazem, rifamycins, cyclosporine, erythromycin, itraconazole, ketoconazole, HIV protease inhibitors, fosamprenavir, and ritonavir), were excluded from the study. Additionally, individuals with a history of secondary dyslipidemia-inducing conditions, such as nephrotic syndrome and hypothyroidism, as well as those with familial hypercholesterolemia, were also excluded from the study.

A post hoc power analysis was conducted based on the observed LDL-C difference of 0.88 mmol/L (β=.877, SE 0.237) and a pooled SD of 0.96 mmol/L. At a significance level of α=.05, the achieved statistical power (1 − β) was estimated at 84%.

### Ethical Considerations

This study was conducted in accordance with the principles of the Declaration of Helsinki and was approved by the Ethics Committee of Biomedical Research at Can Tho University of Medicine and Pharmacy (approval no: 23.052.HV-ĐHYDCT). Prior to enrollment, written informed consent was obtained from all participants after they had been clearly informed of the study’s objectives, procedures, potential risks, and confidentiality safeguards. All data collected were anonymized using unique identifier codes to ensure participant privacy, and no personally identifiable information was included in the dataset or manuscript. Participants received no financial or material compensation for their involvement, as the study was conducted during routine health screenings. Furthermore, no individual-identifiable images or data were presented in the manuscript or supplementary materials; therefore, image consent forms were not required.

### Data Collection

A standardized questionnaire was used to collect clinical and lifestyle data, including age, sex, history of smoking, alcohol abuse, sedentary lifestyle, diabetes mellitus, and hypertension. Anthropometric measurements, including height, weight, and the BMI, were directly measured using standardized procedures. Height was measured to the nearest 0.1 cm, while weight was recorded to the nearest 0.1 kg. The BMI was subsequently calculated as weight in kilograms divided by the square of height in meters (kg/m²). The classification of overweight and obesity was based on the National Institutes of Health and World Health Organization guidelines for the Asian population [19]. Hypertension was defined as previously diagnosed or newly diagnosed according to the 2023 European Society of Cardiology guidelines [20]. Blood pressure measurement procedures were standardized using the 2020 International Society of Hypertension guidelines [21]. Serum urea was quantified using the glutamate dehydrogenase (GLDH) method, which used the enzyme GLDH to measure the reduction of reduced nicotinamide adenine dinucleotide (NADH), a process directly proportional to the urea concentration in the sample. Serum creatinine was measured using the Jaffé kinetic method (the only method available in Vietnam), where creatinine reacts with an alkaline picrate reagent to form a yellow-orange complex [22]. The rate of complex formation is proportional to the creatinine level when compared with a standard. This was performed on the Abbott Architect c4000 automated biochemistry analyzer using the Biolabo reagents from Abbott. Fasting blood glucose and hemoglobin A1c (HbA1c) were tested using the Abbott Architect c4000 automated biochemistry analyzer. Diabetes mellitus was defined as previously diagnosed or newly diagnosed based on the 2023 criteria of the American Diabetes Association or the current use of glucose-lowering medications [23]. After an overnight fast lasting 12 hours, venous blood samples were collected in the morning and processed according to standard laboratory procedures. The serum was separated by centrifugation and used for the determination of lipid profile components, including TC, TGs, HDL-C, and LDL-C. Among these, TC, TGs, and HDL-C were directly measured using enzymatic colorimetric methods on an automated clinical chemistry analyzer, with reagent kits provided with Biolabo reagents. The LDL-C concentration was calculated indirectly using Friedewald’s formula in cases where TG levels were below 400 mg/dL: LDL-C = TC – HDL-C – (TGs/5) [24]. In our study, all participants had TG levels <400 mg/dL, satisfying the prerequisite for the formula’s validity. In addition, serum ApoB levels were also quantified using immunoturbidimetric or chemiluminescent immunoassay methods, depending on the available analytical platform, using Biolabo reagents.

### Genotyping of APOB rs676210 Polymorphism

#### Genomic DNA Extraction

Genomic DNA was extracted from peripheral blood using the Toppure Blood DNA Extraction Kit (ABT), following the manufacturer’s protocol. Briefly, 200 μL of whole blood was mixed with 400 μL of BL buffer and 20 μL of proteinase K, followed by incubation at 72 °C for 10 minutes. After ethanol precipitation, the lysate was transferred to a silica spin column, washed sequentially with Wash Buffer (WB)1 and WB2, and eluted in 50 μL of EB buffer. The purified DNA was stored at –20 °C until use.

#### Real-Time PCR Genotyping

Genotyping of the *APOB* rs676210 polymorphism was performed using a custom-designed allele-specific TaqMan assay. Each 25 μL reaction contained 2.5 μL of 10× polymerase chain reaction (PCR) buffer, 1 μL of primer mix (forward and reverse, 10 μM each), 1 μL of either a 6-carboxyfluorescein (FAM)- or a hexachloro-fluorescein (HEX)-labeled probe (5 μM), 1.5 μL of genomic DNA (10 ng/μL), and nuclease-free water to adjust the volume. The reaction used the EZ PCR Mix (Phu Sa Genomics), a ready-to-use premix containing Taq DNA polymerase, deoxynucleoside triphosphates (dNTPs), and MgCl₂ (final Mg²⁺ concentration=2.0 mM).

Amplification was performed on a CFX Opus 96 Real-Time PCR system (Bio-Rad Laboratories) under the following cycling conditions: initial denaturation at 95 °C for 5 minutes, followed by 35 cycles of denaturation at 95 °C for 25 seconds and annealing/extension at 60 °C for 45 seconds (fluorescence acquisition), and a final extension at 72 °C for 5 minutes. Fluorescence signals were captured and analyzed using CFX Maestro v2.3 software (Bio-Rad Laboratories).

#### Primers and Probes

Primers and allele-specific dual-labeled hydrolysis probes were adapted from Abdulfattah and Al-Awadi [6], with minor sequence modifications to enhance allele specificity. The oligonucleotide sequences were as follows:

- Forward primer: 5′-TGTGTGTGAGATGTGGGGAA-3′
- Reverse primer: 5′-GGGATCTGAAGGTGGAGGAC-3′
- FAM-labeled probe (G allele): 5′-FAM-TCTGGTATGTGAAGGTCAGGA-3′-BHQ1
- HEX-labeled probe (A allele): 5′-HEX-TTCTGATATGTGAAGGTCAGGAAC-3′-BHQ1

All oligonucleotides were synthesized by Macrogen Inc. Primers were desalted, and probes were purified using high-performance liquid chromatography (HPLC).

#### Genotype Interpretation

Allelic discrimination was based on fluorescence threshold detection:

- Homozygous G/G (Pro/Pro): Only the FAM signal exceeded the threshold.
- Homozygous A/A (Leu/Leu): Only the HEX signal exceeded the threshold.
- Heterozygous G/A (Pro/Leu): Both FAM and HEX signals were detected.

No internal control gene was used in the reaction. Duplicate reactions for consistency confirmed genotype calls, and Sanger sequencing was performed on 15% of the total samples to validate the accuracy of genotyping results.

### Data Analysis

Data were checked for completeness and accuracy before analysis. All variables were complete; no missing data were observed. Data collected in this study were encoded and processed using R version 4.3.3 (R Foundation for Statistical Computing), using libraries such as *tidyverse*, *dplyr*, *ggplot2*, *table1*, *compareGroups*, and *pROC* [25]. Data were checked for completeness and accuracy before analysis, with missing or invalid cases excluded. Outliers and abnormal values were detected using frequency plots and basic statistical checks via functions such as *filter()*, *drop_na()*, and *replace_na()*. Categorical variables were numerically coded for statistical processing using the *mutate()* function from *dplyr*, and at least 10% of the data underwent cross-checking using the *sample_n()* function to detect entry errors.

Categorical variables were summarized as frequencies (counts and percentages) using the *table1* package. For continuous variables, data distribution was assessed using the Shapiro-Wilk test, with the *shapiro.test()* function, and values were presented as means (SDs). Prior to statistical analysis, the Kolmogorov-Smirnov and Shapiro-Wilk tests, along with graphical distribution assessments (eg, histograms and quintile-quintile [Q-Q] plots), were used to determine data distribution, guiding the selection of appropriate statistical methods (parametric or nonparametric). When continuous data followed a normal distribution, comparisons between 2 groups were conducted using the Student *t* test, with the *t.test()* function, when the variance assumption was met. Additionally, the Levene test was performed to assess the homogeneity of variances. All continuous lipid variables were confirmed to satisfy the assumptions of normality and homogeneity of variances, validating the use of the Student *t* test for group comparisons. For categorical variables, group comparisons were performed using the chi-square test, with the *chisq.test()* function, when the expected frequency assumptions were satisfied; otherwise, the Fisher exact test, with the *fisher.test()* function, was applied for 2×2 contingency tables, and the Fisher-Freeman-Halton exact test was used for larger contingency tables.

For visualizing continuous data distributions, violin plots were generated using the *ggplot2* package, which provides a clear graphical representation of the data distribution, highlighting the variations in LDL-C and ApoB levels across different genotype groups and allele groups.

The associations between *APOB* rs676210 polymorphism (genotype and allele frequencies) and lipid profile parameters (LDL-C, HDL-C, non–HDL-C, and ApoB) were evaluated using statistical tests. Results are presented in statistical tables, and all tests were 2-tailed, with a significance level of *P*<.05.

## Results

### Participant Details

**Figure 1:**
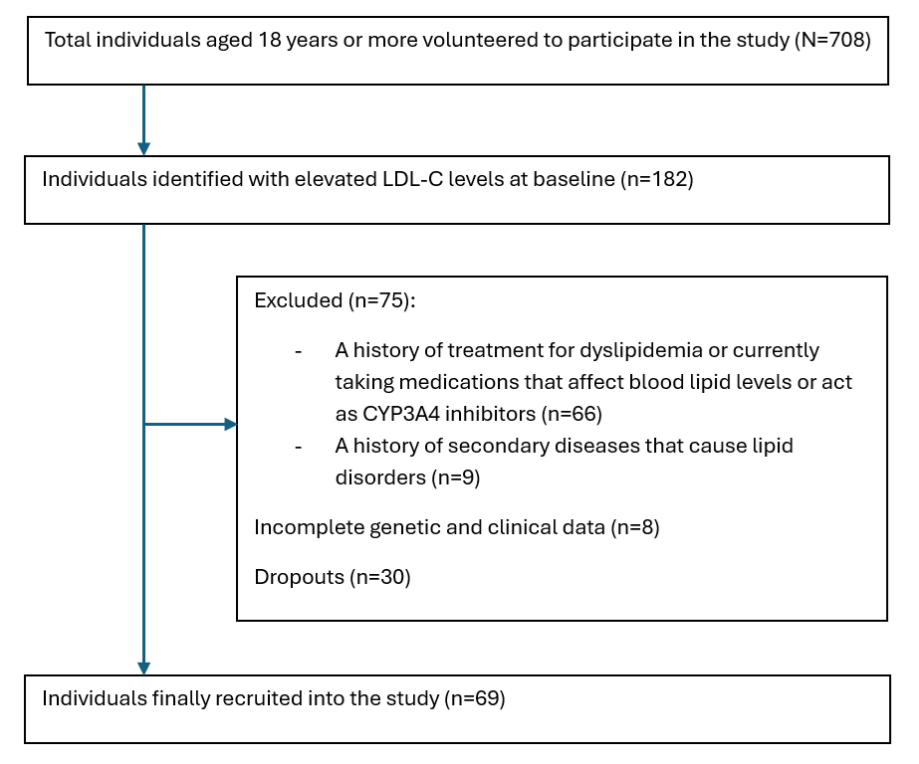
Flow diagram of study participant selection. LDL-C: low-density lipoprotein cholesterol; CYP3A4: cytochrome P450 3A4.

Figure 1 illustrates the flow diagram of participant recruitment and selection. A total of 69 patients with newly diagnosed elevated LDL-C levels were enrolled in the study. Regarding general characteristics, most participants were female, with a female-to-male ratio of approximately 1.38. Most patients were middle-aged, with a mean age of 54.54 (SD 11.64) years, and approximately three-quarters (n=50, 72.5%) of the cohort were overweight or obese. In terms of lifestyle behaviors, about one-quarter (n=16, 23.2%) of participants were current smokers. Concerning medical history, roughly one-third (n=21, 30.4%) had a diagnosis of hypertension, while one-sixth (n=10, 14.5%) had concomitant diabetes mellitus. A relatively high proportion of patients (n=29, 42%) reported a sedentary lifestyle. Additionally, one-sixth (n=12, 17.4%) reported alcohol abuse. With regard to genotype distribution, 32 (46.3%) participants had the AA genotype, 34 (49.3%) had the GA genotype, and 3 (4.4%) had the GG genotype. There were no statistically significant differences in baseline characteristics between the AA and the combined GA+GG genotype groups (*P*<.05), as shown in Table 1.

**Table 1:** Baseline characteristics of study participants stratified by genotype.

Characteristics		Genotype			*P* value
		AA (n=32)	GA+GG (n=37)	Total (N=69)	
**Age (years)**					
	<60, n (%)	23 (71.9)	25 (67.6)	48 (69.6)	.69^a^
	≥60, n (%)	9 (28.1)	12 (32.4)	21 (30.4)	—^b^
	Mean (SD)	53.28 (12.61)	55.62 (10.79)	54.54 (11.64)	.41^c^
**Sex, n (%)**					
	Male	14 (43.8)	15 (40.5)	29 (42)	.79^a^
	Female	18 (56.3)	22 (59.5)	40 (58)	—
Weight (kg), mean (SD)		62.94 (6.06)	60.73 (8.76)	61.75 (7.66)	.24^c^
Height (cm), mean (SD)		160 (7.13)	159.24 (8.42)	159.59 (7.8)	.69^c^
BMI (kg/m^2^), mean (SD)		24.61 (2.09)	23.89 (2.43)	24.23 (2.29)	.19^c^
**Overweight and obesity, n (%)**					
	Yes	25 (78.1)	25 (67.6)	50 (72.5)	.33^a^
	No	7 (21.9)	12 (32.4)	19 (27.5)	—
**Smoking, n (%)**					
	Yes	9 (28.1)	7 (18.9)	16 (23.2)	.37^a^
	No	23 (71.9)	30 (81.1)	53 (76.8)	—
**Alcohol abuse, n (%)**					
	Yes	6 (18.8)	6 (16.2)	12 (17.4)	.78^a^
	No	26 (81.3)	31 (83.8)	57 (82.6)	—
**Sedentary lifestyle, n (%)**					
	Yes	14 (43.8)	15 (40.5)	29 (42.0)	.79^a^
	No	18 (56.3)	22 (59.5)	40 (58.0)	—
**Diabetes mellitus, n (%)**					
	Yes	4 (12.5)	6 (16.2)	10 (14.5)	.74^d^
	No	28 (87.5)	31 (83.8)	59 (85.5)	—
**Hypertension, n (%)**					
	Yes	9 (28.1)	12 (32.4)	21 (30.4)	.70^a^
	No	23 (71.9)	25 (67.6)	48 (69.6)	—

^a^Comparison of the differences are given according to the Pearson chi-square test (statistical significance at *P*<.05).

^b^Not applicable.

^c^Comparison of the differences are given according to the independent samples test (statistical significance at *P*<.05).

^d^Comparison of the differences are given according to the Fisher exact test (statistical significance at *P*<.05).

**Figure 2:**
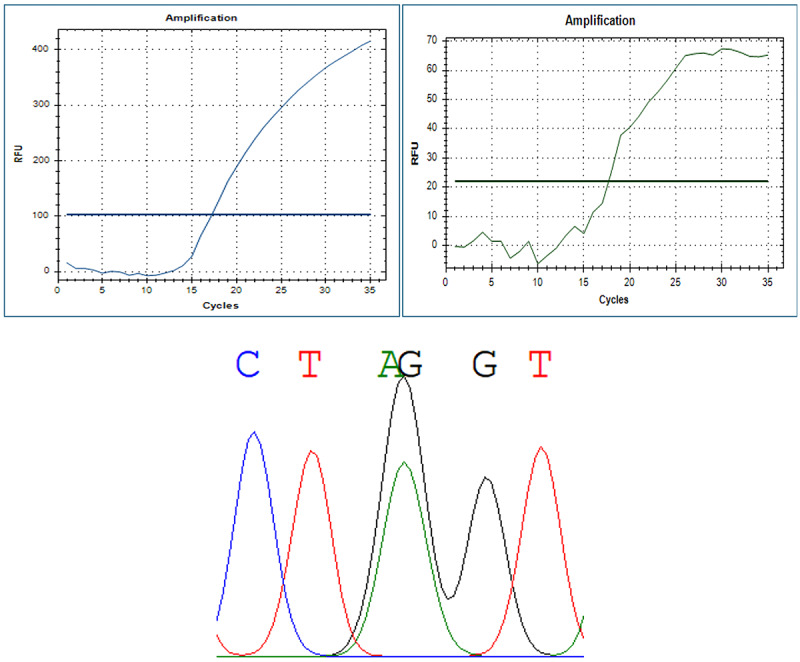
Concordant genotyping results using Sanger sequencing and real-time PCR (patient 24, GA genotype). PCR: polymerase chain reaction; RFU: relative fluorescence units.

Genotyping quality control showed a 100% call rate, with all 10 blinded duplicate samples and 15% of Sanger-validated samples demonstrating 100% concordance. The genotype distribution of the *APOB* rs676210 polymorphism did not deviate significantly from Hardy-Weinberg equilibrium (*P*=.15), supporting assay reliability and population representativeness. Figure 2 shows the concordance between real-time PCR and Sanger sequencing results.

Analysis of the lipid profile among the 69 study participants revealed that individuals with the AA genotype tend to have lower HDL-C levels compared to those with GA and GG genotypes (mean 1.26, SD 0.31, vs mean 1.44, SD 0.39, mmol/L; *P*=.03). Additionally, concentrations of LDL-C, non–HDL-C, and ApoB were significantly higher in the AA genotype group compared to the GA and GG groups (all *P*<.05), as shown in Table 2.

**Table 2:** Lipid profile, ApoBa concentrations, and other indices stratified by genotype.

Parameters		Genotype				*P* value
		AA (n=32)	GA+GG (n=37)	Total (N=69)		
**TC^b^ (mmol/L)**						
	Normal, n (%)	0	4 (10.8)	4 (5.8)	.21^c^	
	Borderline high, n (%)	6 (18.8)	6 (16.2)	12 (17.4)	—^d^	
	High, n (%)	26 (81.3)	27 (73)	53 (76.8)	—	
	Mean (SD)	7.2 (1.18)	6.75 (1.25)	6.96 (1.23)	.14^e^	
**TGs^f^ (mmol/L)**						
	Normal, n (%)	10 (31.3)	13 (35.1)	23 (33.3)	.89^c^	
	Borderline high, n (%)	22 (68.8)	23 (62.2)	45 (65.2)	—	
	High, n (%)	0 (0)	1 (2.7)	1 (1.4)	—	
	Mean (SD)	2.44 (0.67)	2.5 (0.82)	2.47 (0.75)	.73^e^	
**HDL-C^g^ (mmol/L)**						
	Normal, n (%)	26 (81.3)	35 (94.6)	61 (88.4)	.13^c^	
	Decreased, n (%)	6 (18.8)	2 (5.4)	8 (11.6)	—	
	Mean (SD)	1.26 (0.31)	1.44 (0.39)	1.36 (0.37)	.03^e^	
**LDL-C^h^ (mmol/L)**						
	Borderline high, n (%)	4 (12.5)	16 (43.2)	20 (29.0)	.005^c^	
	High, n (%)	28 (87.5)	21 (56.8)	49 (71.0)	—	
	Mean (SD)	5.19 (0.95)	4.37 (0.97)	4.75 (1.04 )	<.001^e^	
Non–HDL-C (mmol/L), mean (SD)		5.94 (1.08)	5.31 (1.22)	5.6 (1.19)	.03^e^	
ApoB (mg/dL), mean (SD)		149.5 (26.3)	136.92 (15.21)	142.75 (21.86)	.02^e^	
Hemoglobin (g/dL), mean (SD)		13.69 (1.26)	13.86 (1.42)	13.78 (1.34)	.59^e^	
HbA1c^i^ (%), mean (SD)		6.05 (1.82)	6.25 (1.75)	6.16 (1.77)	.65^e^	
Glucose (mmol/L), mean (SD)		5.84 (1.84)	5.94 (1.85)	5.9 (1.83)	.83^e^	
Creatinine (μmol/L), mean (SD)		72.3 (16.01)	70.32 (15.48)	71.24 (15.65)	.60^e^	
Urea (mmol/L), mean (SD)		5.2 (1.71)	5.1 (1.57)	5.15 (1.63)	.80^e^	

^a^ApoB: apolipoprotein B.

^b^TC: total cholesterol.

^c^Comparison of the differences are given according to the Fisher-Freeman-Halton exact test (statistical significance at *P*<.05).

^d^Not applicable.

^e^Comparison of the differences are given according to the independent samples test (statistical significance at *P*<.05).

^f^TG: triglyceride.

^g^HDL-C: high-density lipoprotein cholesterol.

^h^LDL-C: low-density lipoprotein cholesterol.

^i^HbA1c: hemoglobin A1c.

**Figure 3:**
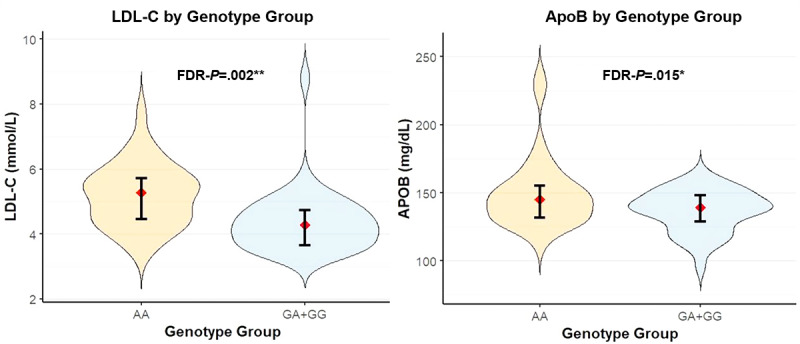
Violin plot of LDL-C and ApoB concentrations by genotypes group. ApoB: apolipoprotein B; FDR-P: false discovery rate–adjusted *P* value; LDL-C: low-density lipoprotein cholesterol.

Figure 3 illustrates the distribution of LDL-C (mmol/L) and ApoB (mg/dL) levels by genotype. Individuals with the AA genotype exhibited higher LDL-C and ApoB concentrations than the GA+GG group. This suggests a potential association between the AA genotype and elevated atherogenic lipid parameters.

To account for potential confounders, multivariable linear regression was performed for lipid parameters by genotype, adjusting for age, sex, the BMI, and diabetes mellitus. As shown in Table 3, associations between the AA genotype and increased LDL-C, non–HDL-C, and ApoB concentrations remained statistically significant after adjustment, while the inverse relationship with HDL-C was attenuated.

**Table 3:** Association between the APOB^a^ rs676210 genotype and lipid profile outcomes: crude and adjusted estimates with multivariable linear regression.

Outcome	Crude estimate *P* value^b^	Model 1^c^ adjusted β (SE; 95% CI)	Model 1 *P* value	Model 2^d^ adjusted β (SE; 95% CI)	Model 2 *P* value	FDR-*P*^e^
HDL-C^f^	.03	–0.157 (0.087; –0.330 to 0.016)	.07	–0.135 (0.088; –0.310 to 0.040)	.13	.07
LDL-C^g^	<.001	0.877 (0.237; 0.403 to 1.352)	<.001	0.922 (0.242; 0.439 to 1.405	<.001	.002
Non–HDL-C	.03	0.673 (0.288; 0.098 to 1.249)	.02	0.745 (0.292; 0.162 to 1.328)	.01	.04
ApoB	.02	11.406 (5.163; 1.092 to 21.720)	.03	12.490 (5.247; 2.004 to 22.975)	.02	.04

^a^*APOB*: apolipoprotein B.

^b^Crude *P* values from unadjusted comparisons between genotype groups.

^c^Model 1: age, sex, and BMI.

^d^Model 2: age, sex, BMI, and diabetes.

^e^FDR-P: false discovery rate–adjusted *P* value calculated for model 1 comparisons to account for multiple testing.

^f^HDL-C: high-density lipoprotein cholesterol.

^g^LDL-C: low-density lipoprotein cholesterol.

Each of the 69 participants contributed 2 alleles, resulting in a total of 138 alleles analyzed for allele-based comparisons. Stratified by genotype, there were no statistically significant differences in baseline characteristics, including age, sex, overweight-obesity status, smoking, alcohol abuse, sedentary lifestyle, diabetes mellitus, and hypertension between allele groups (all *P*>.05), as shown in Table 4.

Allele-based analysis indicated that carriers of the A allele are associated with lower levels of HDL-C and higher levels of LDL-C, non–HDL-C, and ApoB compared to carriers of the G allele (all *P*<.05), as shown in Table 5.

**Table 4:** Baseline characteristics of 69 participants stratified by allele.

Characteristics		Allele				*P* value
		A (n=98)	G (n=40)	Total (N=138)		
**Age group (years)**						
	<60, n (%)	70 (71.4)	26 (65)	96 (69.6)	.46^a^	
	≥60, n (%)	28 (28.6)	14 (35)	42 (30.4)	—^b^	
	Mean (SD)	53.87 (11.96)	56.18 (10.63)	54.54 (11.6)	.29^c^	
**Sex, n (%)**						
	Male	43 (43.9)	15 (37.5)	58 (42)	.49^a^	
	Female	55 (56.1)	25 (62.5)	80 (58)	—	
Weight (kg), mean (SD)		62.6 (6.7)	59.68 (9.3)	61.75 (7.63)	.08^c^	
Height (cm), mean (SD)		160.02 (7.34)	158.55 (8.75)	159.59 (7.77)	.30^c^	
BMI (kg/m^2^), mean (SD)		24.46 (2.15)	23.66 (2.54)	24.23 (2.29)	.06^c^	
**Overweight and obesity, n (%)**						
	Yes	74 (75.5)	26 (65)	100 (72.5)	.21^a^	
	No	24 (24.5)	14 (35)	38 (27.5)	—	
**Smoking, n (%)**						
	Yes	25 (25.5)	7 (17.5)	32 (23.2)	.31^a^	
	No	73 (74.5)	33 (82.5)	106 (76.8)	—	
**Alcohol abuse, n (%)**						
	Yes	18 (18.4)	6 (15.0)	24 (17.4)	.64^a^	
	No	80 (81.6)	34 (85.0)	114 (82.6)	—	
**Sedentary lifestyle, n (%)**						
	Yes	42 (42.9)	16 (40.0)	58 (42.0)	.76^a^	
	No	56 (57.1)	24 (60.0)	80 (58.0)	—	
**Diabetes mellitus, n (%)**						
	Yes	13 (13.3)	7 (17.5)	20 (14.5)	.52^a^	
	No	85 (86.7)	33 (82.5)	118 (85.5)	—	
**Hypertension, n (%)**						
	Yes	29 (29.6)	13 (32.5)	42 (30.4)	.74^a^	
	No	69 (70.4)	27 (67.5)	96 (69.6)	—	

^a^Comparison of the differences are given according to the Pearson chi-square test (statistical significance at *P*<.05).

^b^Not applicable.

^c^Comparison of the differences are given according to the Fisher exact test (statistical significance at *P*<.05).

**Table 5:** Lipid profile, ApoBa concentrations, and other indices stratified by allele.

Parameters		Allele				*P* value
		A (n=98)	G (n=40)	Total (N=138)		
**TC^b^ (mmol/L)**						
	Normal, n (%)	3 (3.1)	5 (12.5)	8 (5.8)	.10^c^	
	Borderline high, n (%)	17 (17.3)	7 (17.5)	24 (17.4)	—^d^	
	High, n (%)	78 (79.6)	28 (70)	106 (76.8)	—	
	Mean (SD)	7.05 (1.2)	6.74 (1.28)	6.96 (1.22)	.18^e^	
**TGs^f^ (mmol/L)**						
	Normal, n (%)	31 (31.6)	15 (37.5)	46 (33.3)	.07^c^	
	Borderline high, n (%)	67 (68.4)	23 (57.5)	90 (65.2)	—	
	High, n (%)	0 (0)	2 (5)	2 (1.4)	—	
	Mean (SD)	2.45 (0.65)	2.51 (0.93)	2.47 (0.74)	.68^e^	
**HDL-C^g^ (mmol/L)**						
	Normal, n (%)	84 (85.7)	38 (95)	122 (88.4)	.15^c^	
	Decreased, n (%)	14 (14.3)	2 (5)	16 (11.6)	—	
	Mean (SD)	1.31 (0.35)	1.46 (0.39)	1.36 (0.37)	.05^e^	
**LDL-C^h^ (mmol/L)**						
	Borderline high, n (%)	22 (22.4)	18 (45.0)	40 (29.0)	.008^c^	
	High, n (%)	76 (77.6)	22 (55.0)	98 (71.0)	—	
	Mean (SD)	4.91 (1.02)	4.36 (0.97)	4.75 (1.03)	.004^e^	
Non–HDL-C (mmol/L), mean (SD)		5.73 (1.14)	5.28 (1.24)	5.6 (1.18)	.04^e^	
ApoB (mg/dL), mean (SD)		145.56 (23.23)	135.88 (15.99)	142.75 (21.78)	.02^e^	
Hemoglobin (g/dL), mean (SD)		13.82 (1.28)	13.7 (1.5)	13.78 (1.34)	.63^e^	
HbA1c^i^ (%), mean (SD)		6.1 (1.75)	6.29 (1.82)	6.16 (1.76)	.58^e^	
Glucose (mmol/L), mean (SD)		5.87 (1.83)	5.96 (1.84)	5.9 (1.83)	.80^e^	
Creatinine (μmol/L), mean (SD)		71.81 (15.83)	69.85 (15.1)	71.24 (15.59)	.51^e^	
Urea (mmol/L), mean (SD)		5.2 (1.66)	5.03 (1.55)	5.15 (1.62)	.58^e^	

^a^ApoB: apolipoprotein B.

^b^TC: total cholesterol.

^c^Comparison of the differences are given according to the Fisher-Freeman-Halton exact test (statistical significance at *P*<.05).

^d^Not applicable.

^e^Comparison of the differences are given according to the independent samples test (statistical significance at *P*<.05).

^f^TG: triglyceride.

^g^HDL-C: high-density lipoprotein cholesterol.

^h^LDL-C: low-density lipoprotein cholesterol.

^i^HbA1c: hemoglobin A1c.

**Figure 4:**
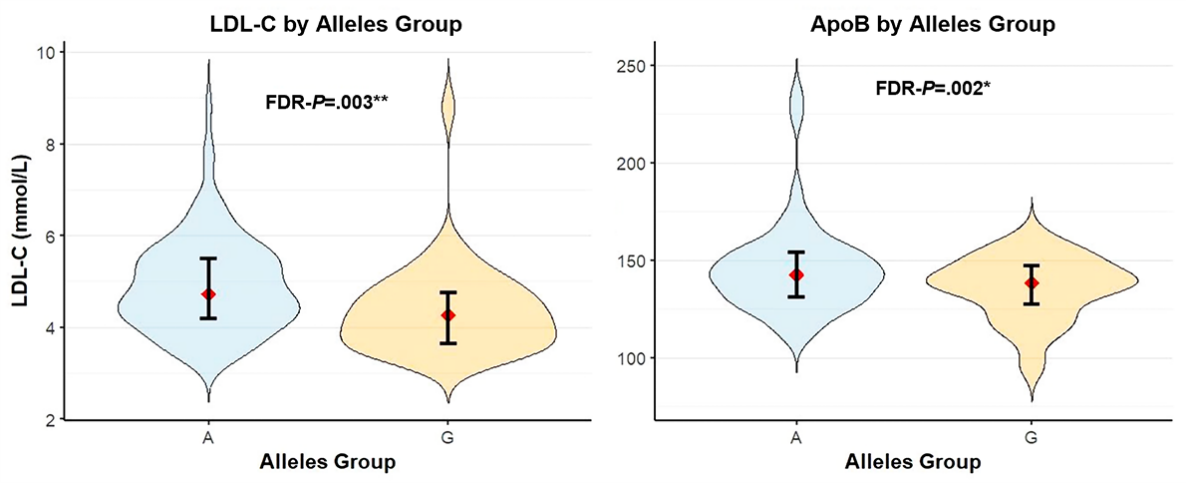
Violin plots of LDL-C and ApoB concentrations by allele. ApoB: apolipoprotein B; FDR-P: false discovery rate–adjusted *P* value; LDL-C: low-density lipoprotein cholesterol.

The violin plots also demonstrated that carriers of allele A exhibit higher concentrations of both LDL-C and ApoB compared to carriers of the G allele (Figure 4).

After adjusting for age, sex, the BMI, and diabetes, the *APOB* rs676210 variant remained significantly associated with increased LDL-C, non–HDL-C, and ApoB levels, as shown in Table 6. The association with HDL-C, however, was attenuated and no longer statistically significant. These results suggest a robust link between the rs676210 genotype and atherogenic lipid parameters, independent of major metabolic confounders.

**Table 6:** Association between *APOB*^a^ rs676210 alleles and lipid profile outcomes: crude and adjusted estimates with multivariable linear regression.

Outcome	Crude estimate *P* value^b^	Model 1^c^ adjusted β (SE; 95% CI)	Model 1 *P* value	Model 2^d^ adjusted β (SE; 95% CI)	Model 2 *P* value	FDR-*P*^e^
HDL-C^f^	.05	–0.091 (0.067; (–0.224 to 0.041)	.17	–0.075 (0.067; (–0.207 to 0.057)	.26	.18
LDL-C^g^	.004	0.606 (0.196; (0.218 to 0.993)	.002	0.621 (0.198; (0.230 to 1.012)	.002	.01
Non–HDL-C	.04	0.512 (0.228; (0.061 to 0.963)	.03	0.549 (0.229; (0.097 to 1.001)	.02	.05
ApoB	.02	7.601 (4.092; (–0.494 to 15.695)	.07	8.202 (4.109; (0.075 to 16.330)	.05	.09

^a^*APOB*: apolipoprotein B.

^b^Crude *P* values from unadjusted comparisons between genotype groups.

^c^Model 1: age, sex, and BMI.

^d^Model 2: age, sex, BMI, and diabetes.

^e^FDR-P: false discovery rate–adjusted *P* value calculated for model 1 comparisons to account for multiple testing.

^f^HDL-C: high-density lipoprotein cholesterol.

^g^LDL-C: low-density lipoprotein cholesterol.

## Discussion

### Principal Findings

In this Vietnamese cohort with newly diagnosed hyper–LDL-C, we observed that individuals carrying the rs676210 AA genotype had a markedly more atherogenic lipid profile than G allele carriers (GA or GG). Specifically, the AA genotype was associated with significantly higher LDL-C, non–HDL-C, and ApoB levels, alongside lower HDL-C. In addition, allele-based analysis revealed that carriers of allele A also had higher LDL-C, non–HDL-C, ApoB levels, and lower HDL-C than G allele carriers.

This pattern suggests that the A allele of rs676210 may predispose to the accumulation of ApoB-containing lipoproteins in circulation. The biological basis for this association likely stems from the functional role of the *APOB* gene variant. Rs676210 causes a Pro2739Leu substitution in the ApoB-100 protein, a change that can alter the protein’s conformation and interactions [6]. Proline is a rigid, helix-breaking residue, whereas leucine is a hydrophobic, helix-forming amino acid [26]; replacing proline with leucine at position 2739 could conceivably influence how ApoB-100 folds or binds to lipid and receptor molecules [27,28]. One consequence of this substitution, as reported in prior studies, is an effect on the susceptibility of LDL particles to oxidation [11,29]. A genome-wide study pinpointed rs676210 as a regulator of oxidized LDL levels, which is noteworthy because oxidized LDL is highly atherogenic and plays a key role in triggering foam cell formation and early atherosclerotic lesions [8]. It is possible that the leucine-encoding A allele produces LDL particles that are less prone to oxidative modification, as one earlier report suggests this variant renders LDL “less” easily oxidized [8]. If LDL is less readily oxidized, it might evade rapid uptake by macrophages and persist longer in circulation, contributing to higher measured LDL-C and ApoB levels. Conversely, the G allele (proline variant) could make LDL particles more susceptible to oxidation and clearance, potentially resulting in relatively lower LDL-C but more oxidative stress per particle. This hypothesis aligns with the notion that rs676210 is “functional” in modifying LDL particle behavior. Of note, our finding that A allele homozygotes have lower HDL-C also points to a broader dysregulation of lipid metabolism associated with this variant, though the mechanism for the HDL effect is unclear. It may be secondary to the remodeling of lipoproteins in an environment of high ApoB lipoprotein concentration. Further biochemical studies are needed to delineate how the Pro2739Leu substitution influences LDL receptor binding, particle clearance rates, or hepatic lipid homeostasis. Nonetheless, the established link between this SNP and LDL oxidation provides a plausible mechanistic bridge from the genotype to the pro-atherogenic lipid phenotype observed in our subjects [7,8,30,31].

The relationship between rs676210 and lipid levels has been examined in several populations, and our results both corroborate and diverge from prior findings. Interestingly, the direction of association observed in our Vietnamese cohort (A allele associated with higher LDL-C and ApoB and lower HDL-C) contrasts with some reports in European ancestry studies. Chasman et al [9], in an extensive genome-wide analysis, noted that the A allele of rs676210 is linked to lower LDL-C and TGs and higher HDL-C. This initially counterintuitive discrepancy highlights the complexity of gene effect modulation by the ethnic and environmental context. In a Chinese population study [11], which focused on myocardial infarction (MI) risk, the G allele of rs676210 (coding for proline at 2739) was identified as the risk variant: Chinese individuals carrying the G allele had higher plasma ApoB levels and an increased risk of MI [11]. This Chinese study also observed a trend toward higher LDL-C in G carriers, although it did not reach statistical significance. These findings imply that in East Asian populations, the G allele may be deleterious, whereas the A (leucine) allele might be relatively protective, consistent with the direction reported by Chasman et al [9] in predominantly European cohorts. By contrast, our findings align more closely with those from a Western Mexican population. Aceves-Ramírez et al [7] reported that Mexican individuals with the AA genotype of rs676210 have significantly elevated odds of acute coronary syndrome, and overall, the A allele confers a higher risk of coronary events compared to allele G (odds ratio [OR] 1.72, *P*<.001). In addition, the A allele frequency was lower in controls (22.5%) than in cases (33%), suggesting the A variant is the risk allele in this population [7]. This parallels our Vietnamese cohort results, where A allele carriers showed a worse lipid profile, consistent with a risk-promoting effect. Notably, allele frequency and linkage disequilibrium patterns for rs676210 vary among ethnic groups. Specifically, East Asians (eg, Han Chinese) have been reported to have a higher A allele frequency (the putatively “normal” allele in those groups) [11], whereas in populations of European or mixed ancestry, the A allele may be the minor variant. Such differences could lead to flip-flopping of which allele appears as “risk” in genetic association studies due to interactions with other genetic loci (epistasis) or environmental factors. Additionally, the context of the study cohorts differs. The Chinese study involved unselected patients with MI and controls [11], whereas ours focused on individuals already flagged for high LDL-C. The latter might represent a subset enriched for genetic hyperlipidemia traits, potentially amplifying the impact of specific alleles. In a population under strong dietary influences or with different baseline risk factor profiles, the effect of rs676210 on measured lipids could manifest differently. For example, high-carbohydrate diets standard in parts of Asia [32-34] might modulate TG-rich VLDL production, interacting with *APOB* variants to influence LDL composition [35,36]. Although speculative, such gene-environment interplay could partly explain why the same SNP shows heterogeneous associations across studies.

Despite these discrepancies, all studies, including ours, reinforce that rs676210 is not a neutral polymorphism but one that influences lipid metabolism in some fashion. Whether the A allele is beneficial or detrimental may depend on the metabolic context. Some have proposed that the leucine variant (A allele) might produce LDL particles less prone to oxidation (potentially reducing inflammatory risk). However, if those particles circulate longer, they could raise LDL-C levels—a trade-off between the quantity and quality of LDL. In contrast, the proline variant (G allele) might shorten LDL residence time at the cost of being more atherogenic per particle. More research is required to resolve these complex dynamics, as well as exceptionally functional assays and population-specific analyses. Our findings contribute to this dialogue by providing data from a Southeast Asian cohort, illustrating that the rs676210-lipid association may parallel that seen in specific Western populations (risk allele A) rather than the pattern reported in East Asians (risk allele G). This underscores the importance of investigating genetic associations within diverse ethnic groups rather than extrapolating findings universally. It should also be noted that participants in our study were identified through annual occupational health examinations, and none had received prior lipid-lowering therapy. Although this allows for the characterization of genotype-phenotype associations in treatment-naive individuals, it may limit generalizability to populations with established CVD or ongoing lipid management.

### Limitations

Our study should be clarified with strengths and limitations. From a clinical and public health perspective, identifying a significant association between *APOB* rs676210 and lipid profiles in Vietnamese patients may carry exploratory implications. First, it highlights a potential genetic marker that could be used to refine risk stratification for dyslipidemia and its downstream consequences. In settings where comprehensive genomic screening is not feasible, testing for a limited panel of impactful SNPs, such as rs676210, could serve as an exploratory tool to flag and identify individuals with a heritable propensity for elevated ApoB and LDL-C. For example, if an individual is known to carry the AA genotype of rs676210, our cross-sectional findings indicate a trend toward a more adverse lipid profile (high LDL-C, high ApoB). These findings may help generate hypotheses regarding whether carriers of the AA genotype are at increased risk for premature ASCVD and could potentially benefit from earlier or more intensive intervention strategies. However, clinical applications remain speculative and would require validation in prospective or interventional studies. Moreover, our sample was drawn from a hospital-based cohort identified through routine occupational health screenings, which may not be fully representative of the general population. Specifically, because all participants were recruited from a hospital-based setting and selected based on newly diagnosed elevated LDL-C without prior statin use, our sample is inherently enriched for individuals with more clinically overt dyslipidemia. This may have amplified the observed genotype-phenotype associations. When compared with findings from 2 recent Vietnamese population-based surveys, the lipid profiles in our cohort appear markedly more atherogenic, underscoring the selection bias and limiting generalizability to the broader community. Caution is therefore warranted when extrapolating these findings beyond the clinical screening context. This is particularly relevant in Vietnam and similar developing contexts, where ASCVD often presents at a relatively young age, and resource-intensive interventions should be targeted to those at most significant risk. Another important implication of our study is the potential for personalized therapy. Evidence suggests that genetic polymorphisms in lipid metabolism genes can influence treatment response. Interestingly, the rs676210 variant has been studied in pharmacogenetic contexts: an Iraqi study demonstrated that patients with the AA genotype experience a greater LDL-C reduction in response to high-dose atorvastatin therapy [6]. Although our study did not involve a treatment intervention, the observed association between the AA genotype and elevated LDL-C levels raises the possibility that individuals carrying this high-risk genotype might benefit more from intensive lipid-lowering therapy, such as high-dose statins. However, this interpretation remains speculative, as our study was not designed to evaluate pharmacogenetic responses. To determine whether the rs676210 polymorphism predicts differential response to statins, a prospective, randomized controlled trial or a genotype-stratified cohort study with pre- and posttreatment lipid measurements would be required. Such a study would help assess whether the AA genotype is not only a marker of increased risk but also a predictor of treatment efficacy. From a hypothesis-generating standpoint, carriers of the A allele might represent a subgroup warranting closer monitoring and, potentially, more intensive therapy—pending validation in longitudinal or interventional studies. It is also worth noting the novelty of our findings. To the best of our knowledge, this is the first report detailing the association of rs676210 with lipid profiles in a Vietnamese cohort. The Vietnamese population has a distinct genetic architecture due to its ethnic background and has been underrepresented in genomic research. Our study contributes preliminary data suggesting that a common *APOB* variant studied in other populations may also be relevant in the Vietnamese context, though confirmation is needed in larger cohorts. Additionally, it is important to note that comprehensive genotype frequency data for the Vietnamese population remain scarce, particularly for variants related to lipid metabolism, such as *APOB* rs676210. This lack of large-scale population-based genetic studies limited our ability to compare the genotype distribution observed in our sample with national reference values or those of neighboring countries. As such, our findings should be interpreted as exploratory and hypothesis generating, reinforcing the need for broader genomic research in this underrepresented population. Lastly, we acknowledge that no sensitivity or subgroup analyses were prespecified, and therefore the findings should be interpreted with appropriate caution.

### Conclusion

In conclusion, this study suggests that the *APOB* rs676210 polymorphism is associated with lipid profile parameters, including LDL-C, non–HDL-C, HDL-C, and ApoB levels, in a Vietnamese population newly diagnosed with elevated LDL-C. Although the study has limitations, including potential selection bias, a moderate sample size, and lack of functional measures (eg, oxidized LDL levels), our findings are biologically coherent and supported by external studies, enhancing confidence in the validity and potential clinical relevance of the association.

## Appendix

Multimedia Appendix 1 Hardy-Weinberg equilibrium (HWE) for the apolipoprotein B (*APOB*) rs676210 variant.

Multimedia Appendix 2 Data collection form (English version).

Multimedia Appendix 3 Data collection form (Vietnamese version).

## Abbreviations

ApoB/*APOB*: apolipoprotein B
ASCVD: atherosclerotic cardiovascular disease
CVD: cardiovascular diseaseHbA1c: hemoglobin A1c
HDL-C: high-density lipoprotein cholesterol
HEX: hexachloro-fluorescein
FAM: 6-carboxyfluorescein
LDL: low-density lipoprotein
LDL-C: low-density lipoprotein cholesterol
MI: myocardial infarction
PCR: polymerase chain reaction
SNP: single nucleotide polymorphism
TC: total cholesterol
TG: triglyceride
VLDL: very low-density lipoprotein
WB: Wash Buffer

## Data Availability

The datasets generated and analyzed during this study are available in the Gen repository on GitHub [37].
