# Supplementary material for: Lipid Profile and Apolipoprotein B Serum Levels in the Vietnamese Population With Newly Diagnosed Elevated Low-Density Lipoprotein Cholesterol and Association With the Single-Nucleotide Variant rs676210: Cross-Sectional Study
Source: JMIR Cardio. 2025 Aug 7;9:e76850. doi: 10.2196/76850 (PMC12371284; doi:10.2196/76850)
Supplement: Multimedia Appendix 1 [file cardio_v9i1e76850_app1.docx]

# Single-SNP Analysis Output

SNP: Genotype

Percentage of typed samples: 69/69 (100%)

| Genotype exact test for Hardy-Weinberg equilibrium (n = 69) | | | | | | |
| --- | --- | --- | --- | --- | --- | --- |
|  | N11 | N12 | N22 | N1 | N2 | *P* |
| All subjects | 32 | 34 | 3 | 98 | 40 | 0.15 |

| Genotype association with response TC (n = 69, crude analysis) | | | | | | |  |
| --- | --- | --- | --- | --- | --- | --- | --- |
| Model | Genotype | n | Response mean (s.e.) | Difference  (95% CI) | P | AIC | NIC |
| Codominant | A/A | 32 | 7.2 (0.21) | 0.00 | 0.32 | 228.8 | 237.8 |
|  | G/A | 34 | 6.77 (0.21) | -0.43  (-1.02 – 0.16) |  |  |  |
|  | G/G | 3 | 6.56 (1.13) | -0.63  (-2.08 – 0.82) |  |  |  |
| Dominant | A/A | 32 | 7.2 (0.21) | 0.00 | 0.13 | 226.9 | 233.6 |
|  | G/A-G/G | 37 | 6.75 (0.2) | -0.45  (-1.02 – 0.13) |  |  |  |
| Recessive | A/A-G/A | 66 | 6.98 (0.15) | 0.00 | 0.57 | 228.9 | 235.6 |
|  | G/G | 3 | 6.56 (1.13) | -0.41  (-1.84 – 1.02) |  |  |  |
| Overdominant | A/A-G/G | 35 | 7.14 (0.21) | 0.00 | 0.21 | 227.6 | 234.3 |
|  | G/A | 34 | 6.77 (0.21) | -0.37  (-0.95 – 0.20) |  |  |  |
| Log-additive |  |  |  | -0.39  (-0.89 – 0.11) | 0.13 | 226.9 | 233.6 |
