# Supplementary material for: Lipid Profile and Apolipoprotein B Serum Levels in the Vietnamese Population With Newly Diagnosed Elevated Low-Density Lipoprotein Cholesterol and Association With the Single-Nucleotide Variant rs676210: Cross-Sectional Study
Source: JMIR Cardio. 2025 Aug 7;9:e76850. doi: 10.2196/76850 (PMC12371284; doi:10.2196/76850)
Supplement: Multimedia Appendix 2 [file cardio_v9i1e76850_app2.pdf]

DATA COLLECTION FORM

LIPID PROFILE AND APOLIPOPROTEIN B SERUM LEVELS IN VIETNAMESE POPULATION WITH NEWLY DIAGNOSED ELEVATED LDL-C AND THE ASSOCIATION WITH THE SINGLE NUCLEOTIDE VARIANT RS676210

Medical Research Number:.............................

A. GENERAL INFORMATION

1. Full name:...

2. Age: ....

3. Sex: ☐ Male ☐ Female

4. Contact Number (if necessary): ....

5. Permanent Address (Province/City): ....

6. Date of Enrollment: ....

B. INCLUSION AND EXCLUSION CRITERIA

1. Has the patient been newly diagnosed with elevated LDL-C? (LDL-C is considered elevated if ≥130 mg/dL or 3.4 mmol/L, based on thresholds established in prior medical literature and the guidelines of the National Cholesterol Education Program - Adult Treatment Panel III [NCEP ATP III], where this level is considered predictive of increased atherosclerotic cardiovascular disease [ASCVD] risk)

   ☐ Yes ☐ No

2. Has the patient ever used or is currently using lipid-lowering medications? (Examples: statins, fibrates, niacin, ezetimibe, high-dose omega-3 fatty acids, etc.)

   ☐ Yes ☐ No

3. Is the patient currently taking any medications that may influence blood lipid levels or CYP3A4 inhibitors? (Examples: corticosteroids, immunosuppressants, oral contraceptives, CYP3A4

inhibitors such as diltiazem, rifamycins, cyclosporine, erythromycin, itraconazole, ketoconazole, HIV protease inhibitors, fosamprenavir, ritonavir, etc.)

☐ Yes ☐ No

4. Does the patient have any known secondary causes of dyslipidemia?

☐ Yes ☐ No

If yes, specify:

- Chronic Kidney Disease (Defined as persistent renal dysfunction ≥3 months, with eGFR <60 mL/min/1.73 m^2 or evidence of chronic kidney damage)?

☐ Yes ☐ No

If yes, specify:

+ CKD Stage: ....

+ eGFR: .... mL/min/1.73 m^2; or

+ Serum Creatinine: .... mg/dL (hoặc µmol/L)

- Nephrotic Syndrome (Defined as proteinuria >3.5 g/day and serum albumin <30 g/L)

☐ Yes ☐ No

If yes, specify:

+ Proteinuria: .... g/day

+ Serum Albumin: ....g/L

- Cirrhosis or Decompensated Liver Disease (Defined as liver stiffness ≥12.5 kPa [FibroScan] or Child-Pugh score ≥B)

☐ Yes ☐ No

If yes, specify:

+ Liver stiffness (FibroScan): ....kPa; or

+ Child-Pugh score: .... points, Grade: ....

- Hypothyroidism (Defined as TSH ≥10 mU/L or current treatment with levothyroxine)

☐ Yes ☐ No

If yes, specify:

+ TSH level: ....mU/L

+ Receiving treatment: ....

5. Does the patient have a known hereditary disorder affecting lipid metabolism?

(Examples: familial hypercholesterolemia, familial hypertriglyceridemia, etc.)

☐ Yes ☐ No

If yes, specify the diagnosis or disease: ....

....

6. Is the patient currently pregnant?

☐ Yes ☐ No

7. Has the patient agreed to participate in the study and signed the informed consent form?

☐ Yes ☐ No

C. CLINICAL CHARACTERISTICS AND LABORATORY TESTS

I. Anthropometric Data

Patient anthropometric measurements were collected via direct assessment at the time of study enrollment, following standardized procedures. The following parameters were recorded:

1. Height:.. cm

2. Weight:... kg

3. Waist circumference: .... cm

4. Body Mass Index (BMI): ....kg/m^2

5. Does the patient have overweight or obesity? (Defined as BMI $\geq$ 23 kg/m^2, according to the classification criteria for Asian populations. BMI is calculated as weight in kilograms divided by the square of height

in meters [kg/m^2]. Classification: underweight [BMI < 18.5 kg/m^2], normal [BMI 18.5–<23 kg/m^2], overweight/obese [BMI ≥ 23 kg/m^2])

☐ Yes ☐ No

6. Blood pressure at the time of assessment:

- Systolic blood pressure:.. mmHg

- Diastolic blood pressure: .... mmHg

II. Lifestyle and Medical History

Information on the patient's lifestyle and medical history was collected as follows:

1. Does the patient smoke cigarettes?

(Defined according to COMMIT study criteria: individuals currently smoking and who have smoked at least 100 cigarettes in their lifetime are considered smokers. Those who have never smoked, or who quit smoking ≥5 years ago, are considered non-smokers.)

☐ Yes ☐ No

2. Does the patient consume alcohol?

(Defined as regular alcohol consumption if ≥1 unit/day [or >10 units/week] for females and ≥2 units/day [or >15 units/week] for males. One unit of alcohol is equivalent to 40 ml spirits, 125 ml wine, or one 330 ml can of beer, according to Vietnamese standards.)

☐ Yes ☐ No

3. Does the patient lead a sedentary lifestyle?

(Assessed using the International Physical Activity Questionnaire [IPAQ]. Adults with physical activity levels below 600 MET-minutes/week are considered sedentary. Activity ≥600 MET-minutes/week is consistent with WHO recommendations for physical activity.)

☐ Yes ☐ No

4. Is there a family history of dyslipidemia?

(Defined as a first-degree relative [father, mother, sibling] previously diagnosed with elevated cholesterol, elevated triglycerides, or mixed dyslipidemia.)

☐ Yes ☐ No

5. Does the patient have hypertension?

(Defined as previously diagnosed hypertension, current use of antihypertensive medication, or newly diagnosed according to the 2023 guidelines of the European Society of Cardiology. Blood pressure measurements were standardized per the 2020 International Society of Hypertension guidelines.)

☐ Yes ☐ No

6. Does the patient have type 2 diabetes mellitus?

(Defined as a prior diagnosis, current use of antidiabetic medications, or newly diagnosed according to the 2023 American Diabetes Association guidelines.)

☐ Yes ☐ No

III. Biochemical Test Results

Blood biochemical data were obtained from fasting venous blood samples collected in the morning after the patient had fasted for at least 8 hours. Blood samples were collected following standardized procedures, centrifuged to separate serum, and analyzed using an automated biochemical analyzer with dedicated reagents. The following parameters were recorded:

1. Total Cholesterol:.. mmol/L
2. Triglyceride:... mmol/L
3. HDL-C (High-Density Lipoprotein Cholesterol): .... mmol/L
4. LDL-C (Low-Density Lipoprotein Cholesterol): .... mmol/L
5. Non-HDL-C (Total Cholesterol – HDL-C): .... mmol/L
6. ApoB (Apolipoprotein B): ....mg/dL
7. Hemoglobin:..g/dL

8. Fasting Plasma Glucose: .... mmol/L

9. HbA1c: .... %

10. Serum Urea: .... mmol/L

11. Serum Creatinine:.. µmol/L

IV. Genotyping Results for APOB Gene Polymorphism rs676210

For genotyping, peripheral blood samples were collected from each participant to analyze the single-nucleotide polymorphism (SNP) rs676210 in the APOB gene. The following data were recorded:

Was the blood sample successfully collected?

☐ Adequate sample collected ☐ Sample not obtained

Patient’s genotyping result (After DNA extraction, the rs676210 genotype was determined using Real-time PCR)

☐ AA ☐ GA ☐ GG

Genetic sequencing result (If available, determined using Sanger sequencing)

☐ AA ☐ GA ☐ GG

V. Additional Notes

(Include any additional relevant information not covered in the sections above, such as complications during sample collection, patient remarks, reasons for failed tests, etc.)

....

....

....

....

STUDY PARTICIPATION CONFIRMATION

I hereby confirm that I have been fully informed of the objectives, content and benefits of this study. I voluntarily agree to participate in the study.

……. Date: ……/……/……

Participant's Signature   Data Collector's Signature
