# Supplementary material for: Lipid Profile and Apolipoprotein B Serum Levels in the Vietnamese Population With Newly Diagnosed Elevated Low-Density Lipoprotein Cholesterol and Association With the Single-Nucleotide Variant rs676210: Cross-Sectional Study
Source: JMIR Cardio. 2025 Aug 7;9:e76850. doi: 10.2196/76850 (PMC12371284; doi:10.2196/76850)
Supplement: Multimedia Appendix 3 [file cardio_v9i1e76850_app3.pdf]

PHIẾU THU THẬP SỐ LIỆU NGHIÊN CỨU
MỐI LIÊN QUAN GIỮA ĐA HÌNH RS676210 CỦA GEN APOB VỚI CÁC THÔNG SỐ LIPID MÁU Ở BỆNH NHÂN CÓ LDL-C TĂNG CAO MỚI PHÁT HIỆN

Mã số bệnh án:............................

A. THÔNG TIN CHUNG

1. Họ và tên: ....

2. Tuổi: ....

3. Giới: ☐ Nam ☐ Nữ

4. Số điện thoại liên hệ (nếu cần):...

5. Địa chỉ thường trú (tỉnh/thành phố): ....

6. Ngày tham gia nghiên cứu: ....

B. TIÊU CHUẨN LỰA CHỌN VÀ LOẠI TRỪ

1. Bệnh nhân có được chẩn đoán tăng LDL-C mới phát hiện không? (LDL-C được xem là tăng khi ≥ 130 mg/dL hoặc 3,4 mmol/L, theo ngưỡng xác định bởi các tài liệu y văn trước đây và khuyến cáo của Chương trình Giáo dục Cholesterol Quốc gia - NCEP ATP III, trong đó mức này được xem là yếu tố dự báo làm tăng nguy cơ bệnh tim mạch xơ vữa [ASCVD])

☐ Có ☐ Không

2. Bệnh nhân đã từng hoặc đang sử dụng thuốc điều trị rối loạn lipid máu? (Ví dụ: statin, fibrate, niacin, ezetimibe, omega-3 liều cao…)

☐ Có ☐ Không

3. Bệnh nhân đang sử dụng thuốc có ảnh hưởng đến nồng độ lipid máu hoặc thuốc ức chế CYP3A4? (Ví dụ: corticosteroids, thuốc ức chế miễn dịch, thuốc tránh thai uống, các chất ức chế CYP3A4 như diltiazem, rifamycins, cyclosporin, erythromycin, itraconazole, ketoconazole, các thuốc ức chế protease HIV, fosamprenavir, ritonavir…)

☐ Có ☐ Không

4. Bệnh nhân có các nguyên nhân có thể gây tăng lipid máu thứ phát?

☐ Có ☐ Không

Nếu có, ghi nhận cụ thể:

- Bệnh nhân có mắc bệnh thận mạn? (Xác định khi bệnh nhân có giảm chức năng thận kéo dài ≥ 3 tháng, dựa vào eGFR < 60 mL/phút/1,73m² hoặc bằng chứng tổn thương thận mạn tính)

☐ Có ☐ Không

Nếu Có, ghi rõ:

+ Giai đoạn bệnh:...

+ Giá trị eGFR:.. mL/phút/1,73m²; hoặc

+ Creatinine máu:... mg/dL (hoặc µmol/L)

- Bệnh nhân có mắc hội chứng thận hư? (Xác định khi protein niệu > 3,5 g/ngày và albumin máu < 30 g/L)

☐ Có ☐ Không

Nếu Có, ghi rõ:

+ Giá trị protein niệu:... g/ngày

+ Giá trị albumin máu:...g/L

- Bệnh nhân có mắc xơ gan hoặc suy gan mất bù? (Xác định khi có chỉ số đàn hồi gan ≥ 12,5 kPa [FibroScan] hoặc điểm Child-Pugh ≥ B)

☐ Có ☐ Không

Nếu Có, ghi rõ:

+ Giá trị chỉ số đàn hồi gan (FibroScan):..kPa; hoặc

+ Điểm Child-Pugh:...................... điểm, phân độ: ....

- Bệnh nhân có mắc suy giáp? (Xác định khi TSH ≥ 10 mU/L hoặc đang điều trị levothyroxine)

☐ Có ☐ Không

Nếu Có, ghi rõ:

+ Giá trị TSH:...mU/L

+ Đang điều trị thuốc: ....

5. Bệnh nhân có mắc bệnh lý di truyền đã biết ảnh hưởng đến chuyển hóa lipid? (Ví dụ: tăng cholesterol máu gia đình, tăng triglyceride máu gia đình...)

☐ Có ☐ Không

Nếu Có, ghi rõ chẩn đoán hoặc tên bệnh lý: ....

....

5. Bệnh nhân hiện đang mang thai?

☐ Có ☐ Không

6. Bệnh nhân có chấp thuận tham gia nghiên cứu và đã ký cam kết đồng thuận?

☐ Có ☐ Không

C. DỮ LIỆU LÂM SÀNG VÀ CẬN LÂM SÀNG

I. Dữ liệu nhân trắc học

Dữ liệu nhân trắc học của bệnh nhân được thu thập thông qua đo lường trực tiếp tại thời điểm tham gia nghiên cứu, tuân thủ theo quy trình chuẩn hóa. Các số liệu cần thu thập bao gồm:

1. Chiều cao:.. cm

2. Cân nặng: .... kg

3. Vòng eo: .... cm

4. Chỉ số khối cơ thể (BMI): ....kg/m²

5. Bệnh nhân có tình trạng thừa cân hoặc béo phì? (Xác định khi BMI đạt từ 23 kg/m² trở lên, theo tiêu chuẩn phân loại dành cho người châu Á. Cụ thể, BMI được tính bằng cân nặng (kg) chia cho bình phương chiều cao (m²). Theo phân loại gầy (BMI < 18,5 kg/m²), bình thường (BMI từ 18,5 đến < 23 kg/m²) và thừa cân - béo phì (BMI ≥ 23 kg/m²)

☐ Có ☐ Không

6. Huyết áp đo tại thời điểm khảo sát:

- Tâm thu:.. mmHg

- Tâm trương: .... mmHg

II. Lối sống và tiền sử

Thông tin về lối sống và tiền sử của bệnh nhân được thu thập bao gồm:

1. Bệnh nhân có hút thuốc lá? (Xác định theo tiêu chuẩn của nghiên cứu COMMIT. Người đang có hút thuốc lá và đã hút ít nhất 100 điếu trở lên được xem là có hút thuốc lá. Người chưa bao giờ hút thuốc lá hoặc có hút nhưng đã nghỉ hút ít nhất là 5 năm trở lại đây được xem là không hút thuốc lá)

☐ Có ☐ Không

2. Bệnh nhân có uống rượu bia? (Tiêu thụ ≥ 1 đơn vị /ngày đối với nữ [hoặc > 10 đơn vị/tuần] và ≥ 2 đơn vị/ngày [hoặc > 15 đơn vị/tuần] đối với nam được xem là có uống rượu bia thường xuyên, 1 đơn vị rượu tương đương 40 ml rượu mạnh, 125 ml rượu vang hoặc 1 lon bia 330 ml theo tiêu chuẩn Việt Nam)

☐ Có ☐ Không

3. Bệnh nhân có lối sống ít vận động? (Đánh giá dựa trên bảng câu hỏi hoạt động thể lực quốc tế - IPAQ. Người trưởng thành có mức hoạt động thể lực dưới 600 MET-phút mỗi tuần được xem là ít vận động. Ngược lại, mức hoạt động từ 600 MET-phút/tuần trở lên được coi là đáp ứng khuyến cáo của Tổ chức Y tế Thế giới về vận động thể lực)

☐ Có ☐ Không

4. Tiền sử gia đình có rối loạn lipid máu? (Xác định khi có người thân trực hệ [cha, mẹ, anh/chị/em ruột] được chẩn đoán tăng cholesterol, tăng triglyceride, hoặc mắc rối loạn lipid hỗn hợp trước đây)

☐ Có ☐ Không

5. Bệnh nhân có mắc tăng huyết áp? (Xác định khi bệnh nhân đã được chẩn đoán tăng huyết áp trước đó hoặc đang điều trị bằng thuốc hạ áp hoặc mới được chẩn

đoán theo hướng dẫn của Hiệp hội Tim mạch Châu Âu năm 2023, các quy trình đo huyết áp đã được chuẩn hóa theo hướng dẫn của Hiệp hội Tăng huyết áp Quốc tế năm 2020)

☐ Có ☐ Không

6. Bệnh nhân có mắc đái tháo đường típ 2? (Xác định khi đã được chẩn đoán trước đó hoặc đang điều trị với thuốc kiểm soát đường huyết hoặc mới được chẩn đoán theo hướng dẫn của Hiệp hội Đái tháo đường Hoa Kỳ 2023)

☐ Có ☐ Không

III. Kết quả xét nghiệm sinh hóa

Dữ liệu hóa sinh máu của bệnh nhân được thu thập thông qua xét nghiệm máu tĩnh mạch vào buổi sáng, sau khi bệnh nhân đã nhịn ăn ít nhất 8 giờ. Mẫu máu được lấy theo quy trình chuẩn, xử lý ly tâm để tách huyết thanh và phân tích bằng hệ thống máy sinh hóa tự động với các bộ hóa chất chuyên dụng. Các số liệu cần thu thập bao gồm:

1. Cholesterol toàn phần (Total Cholesterol):...................................... mmol/L
2. Triglyceride:... mmol/L
3. HDL-C (High-Density Lipoprotein Cholesterol): .... mmol/L
4. LDL-C (Low-Density Lipoprotein Cholesterol): .... mmol/L
5. Non-HDL-C (Total Cholesterol – HDL-C): .... mmol/L
6. ApoB (Apolipoprotein B): ....mg/dL
7. Hemoglobin:..g/dL
8. Glucose máu đói (Fasting Plasma Glucose): .... mmol/L
9. HbA1c: ....%
10. Ure:.. mmol/L
11. Creatinine:.. µmol/L

IV. Kết quả xét nghiệm đa hình gen rs676210 (gen APOB)

Để phân tích kiểu gen, sử dụng mẫu máu ngoại vi được lấy từ mỗi đối tượng nghiên cứu nhằm xét nghiệm đa hình đơn nucleotide rs676210 thuộc gen APOB. Các thông tin cần thu thập bao gồm:

Tình trạng thu nhận mẫu máu của bệnh nhân?

☐ Lấy đủ mẫu ☐ Không lấy được mẫu

Kết quả phân tích gen của bệnh nhân? (Sau khi chiết tách DNA, kiểu gen rs676210 được xác định bằng phương pháp Realtime-PCR)

☐ AA ☐ GA ☐ GG

Kết quả giải trình tự gen của bệnh nhân? (Nếu có, giải trình tự bằng phương pháp Sanger)

☐ AA ☐ GA ☐ GG

V. Ghi chú khác

(Điền các thông tin bổ sung ngoài các mục đã liệt kê ở trên – nếu có, ví dụ: biến chứng trong quá trình thu thập mẫu, ghi chú từ bệnh nhân, lý do không lấy được xét nghiệm…)

....

....

....

....

XÁC NHẬN THAM GIA NGHIÊN CỨU

Tôi xác nhận rằng tôi đã được giải thích đầy đủ về mục tiêu, nội dung, lợi ích và nguy cơ của nghiên cứu. Tôi đồng ý tham gia nghiên cứu trên tinh thần tự nguyện.

……. Ngày: ……/……/……

Chữ ký đối tượng nghiên cứu Chữ ký người thu thập số liệu
